# Bioeconomic production of high-quality chitobiose from chitin food wastes using an in-house chitinase from *Vibrio campbellii*

**DOI:** 10.1186/s40643-022-00574-8

**Published:** 2022-08-20

**Authors:** Reeba Thomas, Tamo Fukamizo, Wipa Suginta

**Affiliations:** School of Biomolecular Science and Engineering (BSE), Vidyasirimedhi Institute of Science and Technology (VISTEC), Payupnai, Wangchan District, Rayong, 21210 Thailand

**Keywords:** Chitin, Chitinase, Chitooligosaccharides, Bioeconomy, Bioconversion, *Vibrio* species, Chitin recycling

## Abstract

Marine *Vibrio* species are natural degraders of chitin and usually secrete high levels of chitinolytic enzymes to digest recalcitrant chitin to chitooligosaccharides. This study used an endochitinase (*Vh*ChiA) from *Vibrio campbellii* to produce high-quality chitobiose from crustacean chitins. The enzyme was shown to be fully active and stable over 24 h when BSA was used as an additive. When different chitin sources were tested, *Vh*ChiA preferentially digested shrimp and squid (α) chitins compared to crab (β) chitin and did not utilize non-chitin substrates. The overall yields of chitobiose obtained from small-scale production using a single-step reaction was 96% from shrimp, and 91% from squid pen and crab-shell chitins. Larger-scale production yielded 200 mg of chitobiose, with > 99% purity after a desalting and purification step using preparative HPLC. In conclusion, we report the employment of an in-house produced chitinase as an effective biocatalyst to rapidly convert chitin food wastes to chitobiose, in a quantity and quality suitable for use in research and commercial purposes. Chitobiose production by this economical and eco-friendly approach can be easily scaled up to obtain multi-gram quantities of chitobiose for chemo-enzymic synthesis of rare chitooligosaccharide derivatives and long chain chitooligosaccharides, as well as preparation of sugar-based functionalized nanomaterials.

**Graphical Abstract:**

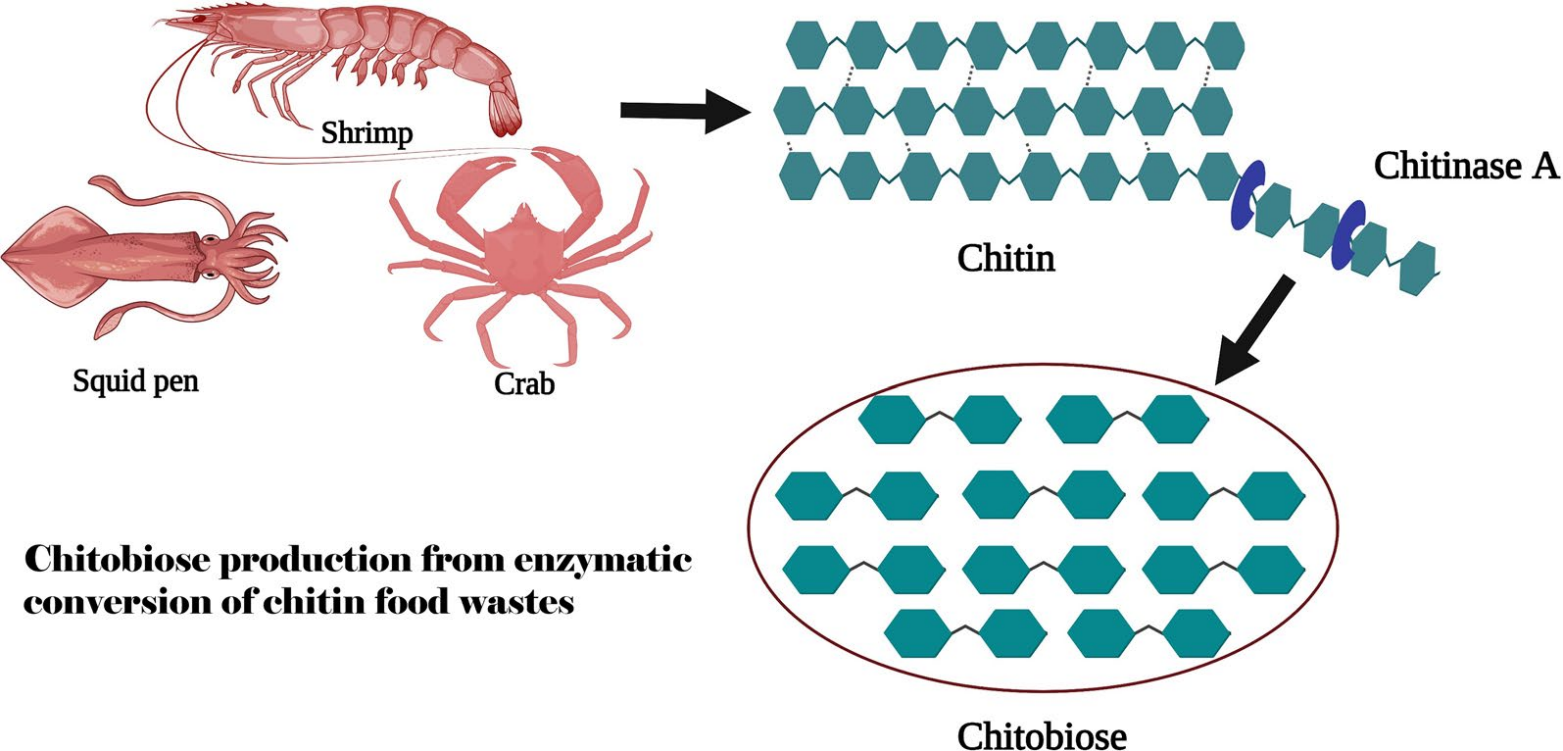

**Supplementary Information:**

The online version contains supplementary material available at 10.1186/s40643-022-00574-8.

## Introduction

Chitin, one of the most abundant structural polysaccharides in nature, is composed of repeated units of *N*-acetylglucosamine (GlcNAc) linked by *β*-(1,4)-glycosidic bonds. It is predominantly found in the cell walls of fungi and the exoskeletons of invertebrates, including insects and crustaceans. Recalcitrant chitin is insoluble and for dissolution it requires solubilizing agents to break intermolecular hydrogen bonding. Structurally, chitin is classified into α-, β- or γ-forms (Jang et al. 2004), depending on the internal chain arrangements. α-Chitin is found mainly in shrimp- and crab-shells. The polysaccharide chains of α-chitin fold in an anti-parallel fashion, and each chain is held tightly to its adjacent chain by a number of interchain and intrachain hydrogen bonds (Minke and Blackwell 1978). β-Chitin is found in squid pens, in which the neighboring chitin chains are aligned in parallel, resulting in less densely packed polysaccharide sheets, due to the lack of hydrogen bonds between adjacent sheets (Gardner and Blackwell 1974). γ-Chitin is the least common natural form; it contains a mixture of parallel and anti-parallel chitin chains with X-ray diffraction patterns similar to those of both α- and β-chitins (Jang et al. 2004). These different packing patterns are responsible for the different physicochemical properties of the chitin forms: α-chitin is the most tightly packed and is insoluble in water, while β- and γ-chitins are more water accessible, with more flexible structures (Cohen 2010).

Hydrolysis of chitin yields chitooligosaccharides (CHOs), which are water-soluble reaction products that can be used in industrial, pharmaceutical and biotechnological applications (Zhao 2019). CHOs are generally temperature- and pH-tolerant, which is important in maintaining their antifungal, insecticidal, anti-microbial, anti-tumor and anti-oxidative activities, as well as their roles as drug carriers (Liaqat and Eltem 2018; Zhao 2019). CHOs can be produced by physical, chemical or enzymic processes (Liaqat and Eltem 2018). Traditionally, the initial preparation of chitin includes physical methods, such as ultrasonication, swirling cavitation and γ-irradiation (Baxter et al. 2005; Zainol et al. 2009; Wu et al. 2014), followed by a chemical process, which includes three steps: demineralization, deproteinization and decoloration (Hamed et al. 2016). These steps involve the use of concentrated hydrochloric acid and sodium hydroxide, which are environmentally hazardous and make CHOs unsuitable for human consumption because of the toxicity of contaminated by-products. CHO production by chemical methods is limited to small milligram yields (Je and Kim 2012) and usually a mixture of CHOs of different chain lengths is produced (Aam et al. 2010). Therefore, time-consuming and labor-intensive techniques, usually a combination of gel filtration chromatography (Sørbotten et al. 2005), ultrafiltration (Lopatin et al. 2009) and ion-exchange chromatography (Haebel et al. 2007), are required for CHO separation and purification.

From an environmental point of view, enzymic approaches have gained attention because the hydrolytic reaction takes place under mild conditions, usually at ambient temperature and physiological pH and in a non-toxic buffered system, making the products environment-friendly and safe for human uses (Sinha et al. 2016; Sheldon and Woodley 2018). Enzymic depolymerization of chitin involves chitinases (EC 3.2.1.14), which hydrolyze the *β*-(1,4)-glycosidic bonds that link the GlcNAc units in a chitin chain, generating (GlcNAc)_2_ (chitobiose) as the final product. Chitinases are classified as families GH-18 and GH-19 in the Carbohydrate-Active enZYmes database (CAZy) (Cantarel et al. 2009; Davies and Henrissat 1995; Henrissat and Bairoch 1996), according to their amino acid sequences and their mode of action (Henrissat 1991; Henrissat and Bairocht 1993). GH-18 chitinases are distributed in various organisms, including bacteria, insects, fungi, higher plants and animals (Chen et al. 2020). Chitinolytic bacteria are a particular class of bacteria that grow on chitinous surfaces by secreting different classes of chitinolytic enzymes, including endochitinases (E.C. 3.2.1.14), exochitinases or chitobiase (E.C. 3.2.1.29) and *N*-acetylglucosaminidases (E.C. 3.2.1.30) (Cohen-Kupiec and Chet 1998) to degrade chitin to CHOs that serve as the bacteria’s carbon and nitrogen sources (Le and Yang 2018). Marine *Vibrio* species are strictly chitinolytic bacteria, owing to their high abundance in aquatic habitats. The chitin catabolic machinery was shown to be highly active in marine *Vibrio* bacteria, enabling them to utilize chitin as the major source of nutrients (Keyhani and Roseman 1999; Suginta 2007; Suginta et al. 2004, and Suginta et al. 2000).

Aquaculture is a major source of employment and world gross income. The Food and Agricultural Organization of the United Nation (FAO) *(*https://www.fao.org/state-of-fisheries-aquaculture*)* reports that global seafood production is around 170 million metric tons (live weight) per annum, with a world trade volume for marine crustaceans of *approx.* 8.4 million metric tons reported for 2017 (https://www.persistencemarketresearch.com/market-research/crustacean-market.asp). Thailand is one such country, whose economy depends on agriculture, with the annual harvest from aquaculture in Thailand officially estimated to be an average production of 3.4 ± 0.5 metric tons per year from 1999 to 2015, with Thailand’s shrimp production being around 0.5—0.6 metric tons per year (https://www4.fisheries.go.th/local/index.php/main/site/strategy-stat; Sriboonchitta et al. 2001). Seafood processing is a major industry, with Thailand’s total export revenue from the seafood sector estimated to be approximately 7 billion euros annually. Approximately six to eight million tons of seafood wastes are generated each year from crab, shrimp and lobster shells, causing environmental concerns associated with the disposal of accumulating seafood wastes along coastal areas across the world. Taken that these crustacean shells are rich in the polysaccharide chitin (15–40% of total weight), biodegradation of chitin food wastes to added-value CHOs not only offers effective, eco-friendly waste managements, but the sugar products in itself has a high market value. High biocompatibility and bioactive properties of CHOs products enable them to be used in various fields of agriculture, biotechnology and biomedicine (Yan and Chen 2015). It is therefore an attractive idea to exploit enzyme-based technology to transform chitin-containing seafood wastes into bioactive chitooligosaccharides of much greater value for commercial uses.

We previously reported the identification and detailed functional and structural characterization of a GH-18 endochitinase, *Vh*ChiA, from the marine bioluminescent chitinolytic bacterium *Vibrio campbellii* (formerly classified as *V. harveyi*) ( Lin et al. 2009; Songsiriritthigul et al. 2008; Suginta et al. 2000, 2004, 2010). *Vh*ChiA was highly active against chitin polysaccharides (Suginta et al. 2004, 2005), generating various CHO intermediates, with chitobiose as the final product. The aim of this study is to employ in-house chitinase, which is a stable enzyme and could be conveniently produced in our laboratory as a biocatalyst for chitobiose production from chitin food wastes generated by seafood processing factories along the coastline of the Gulf of Thailand. The single-step enzymic reaction described here offers a fast production of chitobiose of analytical grade (> 99% purity), and gram quantities of chitobiose could potentially be obtained in a small-scale bioreactor. In addition, this bio-innovative technology using an in-house produced chitinase for chitin recycling offers inexpensive yet highly effective biowaste management, as well as a stimulus to sustainable bioeconomy that can be adopted in any country with an economy that relies on fisheries and the seafood processing industries.

## Materials and methods

### Preparation of chitin

Chitin used in this study was obtained from three different sources: shrimp shells (Marine Bio Resources Co., Ltd., Thailand), crab shells (Practical grade, Sigma-Aldrich, Germany), and squid pens (Marine Bio Resources Co., Ltd., Thailand). Colloidal chitin was prepared by acid treatment using the protocol described previously (Murthy and Bleakley 2012). In brief, 20 grams of chitin flakes in a 1000-mL glass beaker were added into 150 mL of 12 M HCl, added slowly with continuous stirring, and then stirred at 25 °C overnight. The supernatant containing HCl was then discarded after centrifugation at 3,924 x*g* for 30—60 min at 4 °C and the chitin pellet was washed thoroughly with ice-cold distilled water (DI) until the pH of the chitin suspension was close to 7.0. The acid-treated chitin (at this stage called colloidal chitin) was air-dried in an oven at 60 °C, ground in a mortar and used as substrate for the preparation of chitobiose by *Vh*ChiA.

### Expression and purification of *Vh*ChiA

The DNA fragment encoding full-length *Vh*ChiA was cloned into the pQE60 expression vector and expressed in *E. coli* M15 cells at a high level as described previously **(**Suginta et al. 2004). For recombinant expression, the transformed cells were grown at 37 °C in LB medium containing a final concentration of 100 μg.mL^−1^ ampicillin and 50 μg.mL^−1^ kanamycin until the *OD*_600_ of the culture reached 0.6–0.8. The cell culture was then cooled on ice before chitinase expression was induced by the addition of isopropylthio-β-D-galactoside (IPTG) to a final concentration of 0.2 mM. Cell growth was continued at 25 °C for an additional 18 h, and the cell pellet was collected by centrifugation at 3,924x* g* at 4 °C for 30 min. The pellet was resuspended in freshly prepared lysis buffer (20 mM Tris HCl, pH 8.0, 150 mM NaCl, 1 mM PMSF and 1 mg.mL^−1^ egg white lysozyme) and further lysed on ice using an ultrasonic processor (Cole-Parmer, Vernon Hills, Illinois, USA) with a 1-cm diameter probe (Amp: 30%, Pulse ON: 20 s, OFF: 40 s, Timer: 40 min). Unbroken cells and cell debris were removed by centrifugation at 16,773x *g* for 45 min at 4 °C. The supernatant was immediately applied to a Ni-charged Resin affinity column (GenScript USA, Inc. Piscataway, NJ08854, USA) and chromatography was carried out under gravity at 4 °C; after loading, the column was equilibrated with equilibration buffer (20 mM Tris–HCl, pH 8.0, 150 mM NaCl) containing 20 mM imidazole. The bound proteins were eluted with 150 mM imidazole in equilibration buffer. Eluted fractions (5 mL) were collected, and each fraction was analyzed by 12% SDS-PAGE to confirm purity. Fractions containing chitinase were pooled and then subjected to multiple rounds of dialysis (Snake Skin™ Dialysis Tubing, 3.5 K MWCO, 35-mm dry I.D, Thermoscientific, Meridian Rd, Rockford, U.S.A) in equilibration buffer for the complete removal of imidazole. The purified protein was concentrated using an Amicon Ultra-15 centrifugal filter unit, 30 K-cut off (Merck Millipore, Tullagreen, Cork, Ireland) to 19 mg.mL^−1^, then aliquots were stored at -80 °C until use.

### Chitinase activity assay

Chitinase activity was determined by a colorimetric assay using *p*NP-(GlcNAc)_2_ (Megazyme, Neogen, Ireland) as substrate**.** The assay was carried out in a 96-well microtiter plate, with a 100-μL reaction mixture containing various concentrations of *p*NP-(GlcNAc)_2_ (0, 6.25, 12.5, 25, 50, 100, 250, 500 μM), the protein sample (0.2 μg.μL^−1^) and 0.1 M sodium acetate buffer, pH 5.5. The mixture was incubated at 30 °C for 10 min with constant agitation, and the reaction terminated by the addition of 100 μL of 3 M sodium carbonate (Na_2_CO_3_). The amount of liberated *p*NP was determined spectrophotometrically at 405 nm in a microtiter plate reader (ThermoFisher scientific, Ratastie 2, Finland) and a standard curve was obtained with *p*NP solution (0 to 50 nmol). One unit (U) of the chitinase activity is defined as the amount of enzyme required for the liberation of 1 nmol of *p*NP per min. Chitinase activity assays were usually carried out in triplicate, unless otherwise stated.

### Enzyme stability study

*Vh*ChiA was tested for stability at different reaction times. The reaction mixture (100 μL) in a microplate reader (ThermoFisher scientific, Ratastie 2, Finland), containing *p*NP-(GlcNAc)_2_ (500 μM), *Vh*ChiA (10 U) and 0.1 M sodium acetate buffer, pH 5.5, was incubated at 30 °C with constant agitation. At each time point the reaction mixture was withdrawn and flash-frozen in liquid N_2_. The chitinase activity was determined as described above using *p*NP-(GlcNAc)_2_ as the substrate. The effect of bovine serum albumin (BSA) on the enzyme stability was also tested. Reactions were carried out in the absence and presence of 4 μg per well of BSA (ACROS, New Jersey, USA) and the residual chitinase activities under the two different conditions were examined at different times of reaction from 0 to 24 h.

### Substrate specificity

The specificity of *Vh*ChiA was studied using different polysaccharide substrates including mannan, partially deacetylated chitin (chitosan), Avicel® microcrystalline cellulose and squid pen chitin. The time-courses of degradation of these polysaccharides were determined and the reaction products analyzed by thin layer chromatography (TLC) (Tanaka et al. 1999). The reaction mixture, consisting of 2 mg of each polysaccharide, 40 U (8 μg) of *Vh*ChiA and BSA (16 μg or 1:2 ratio of chitinase to BSA) in 2 mL of 0.1 M sodium acetate buffer, pH 5.5 was incubated at 30 °C with continuous agitation. For product analysis, the TLC silica plate (TLC silica gel 60 F254, aluminum sheets, Merck, Germany) was pre-heated at 60 °C prior to sample application to remove any absorbed moisture. Aliquots of the reaction mixture withdrawn at different incubation times from 0 min to 24 h were applied six times (1 μL each) to the silica plate and chromatographed four times (1 h each), followed by spraying with aniline–diphenylamine reagent and the plate was heated until visible spots were observed. The mobile phase used for TLC contained butanol:methanol:28% ammonia solution:water (10:8:4:2 *v/v*). The substrate specificity of *Vh*ChiA was also tested with different chitooligosaccharides (GlcNAc)_2–6_ (a final concentration of 1.5 mM of each CHO, prepared in distilled water) using the protocol described above (data not shown).

### A small-scale production of chitobiose and product analysis by TLC

Chitobiose production was carried out in two steps: first, pretreatment of crystalline chitin with HCl and second, enzymic hydrolysis. For small-scale production (2 mL), chitin from squid pens or shrimp or crab shells (5 mg of each) was incubated with 100 U (20 μg) of enzyme and 40 μg of BSA as a stabilizer in 0.1 M sodium acetate buffer at 30 °C and hydrolysis was carried out for 24 h. Aliquots of 120 μL were withdrawn after different time intervals (0, 2.5, 5.0, 10.0, 30.0 min, and 1.0, 16, 24 h) and the reaction was terminated by heating in a block at 98 °C for 5 min followed by centrifugation at 13,817 x*g* at 4 °C for 20 min. The degradation products were then analyzed by TLC, following the method described earlier.

### Quantitative analysis by HPLC

Chitooligosaccharide products generated from chitin hydrolysis by *Vh*ChiA were determined with a gel-filtration column connected to an HPLC system. Each aliquot (20 μL) obtained from the reaction mixture at various reaction times was injected into a TSK Gel G2000 PW column (7.5 mm ∅ × 30 cm L) connected to a high-performance liquid chromatography (HPLC) system (Shimadzu, High Performance Liquid Chromatography Prominence – I LC – 2030 series, Nexera -I LC 2040 series) (Shimadzu Bara Scientific Co., LTD, Bangkok Thailand). Deionized water was used as the mobile phase, and the column was operated isocratically at 150 psi with a flow rate of 0.1 mL.min^−1^ and temperature 25 ± 1 °C. The products were monitored by absorption at 200 nm (*A*_200_) using a photodiode array detector (PDA). The data were extracted and plotted in GraphPad Prism *v*.5.01 and the concentrations of the hydrolytic products were calculated from the standard curves obtained with the mixture of CHOs, (GlcNAc)_1–4_.

### Hundred-milligram scale production of chitobiose

Larger scale production of chitobiose was carried out in an Erlenmeyer flask containing 1 g of dried shrimp colloidal chitin, 20,000 U (4 mg) of *Vh*ChiA and 8 mg of BSA in 0.1 M sodium acetate buffer, pH 5.5 in total volume of 1L.After incubation for 24 h at 30 °C, the reaction mixture was centrifuged at 2359 g for 40 min at 4 °C to remove the remaining chitin substrate from the reaction mixture and concentrated using a centrifugal concentrator (Amicon Ultra-15 centrifugal filter unit, 30 K-cut off (Merck Millipore, Tullagreen, Cork, Ireland) at 4129 x*g* for 30 min at 4 °C. The solution was then dried in a rotavapor (IKA RV 10 digital Rotary Evaporator line, IKA® Works (Thailand) Co. Ltd., Bangkok, Thailand) supported with an IKA MVP10 basic compact vacuum pump and Model K-015 chiller Circulator (heating temperature 40 °C, cooling temperature 2 °C, pressure 0.29 psi, rotation at 40 rpm). The completely dried products were dissolved in 10 mL deionized water and passed through a gel filtration column (2.1 cm × 160 cm) packed with cellulose beads (Cellufine, Lot No. R2407, JNC Corporation, Japan), and the eluted fractions were collected by gravity using a fraction collector (Bio Rad Model 21,100, USA). Fractions with ultraviolet absorption at 200 nm were analyzed by TLC and the fractions corresponding to chitobiose were then pooled and concentrated in a rotavapor, then freeze-dried. Chitobiose in powder form was stored at room temperature (25 ± 2 °C) in vacuo.

### Purification and salt elimination of chitobiose by preparative HPLC

In the final step, salt was eliminated from chitobiose to obtain highly purified chitobiose. One gram of the chitobiose/salt powder obtained from Cellufine gel filtration column was dissolved in 3 mL of deionized water. Then, aliquots of 45 µL of the solubilized sample were injected multiple times into an Asahipak NH_2_P-50 10E preparative column (10.0 mm × 250 mm, Shodex China Co., Ltd) connected to a Shimadzu HPLC system. The HPLC separation was conducted at a temperature of 25 ± 1 °C under a pressure of 2000 psi with a flow rate of 1.0 mL.min^−1^ and elution by a gradient of acetonitrile:water (70:30 *v/v*) as the mobile phase. The separated products were detected at 200 nm using a PDA detector. Eluted fractions (6.0 mL each) corresponding to the chitobiose peak were pooled and concentrated in a Rotavapor followed by freeze-drying and the purified chitobiose powder was stored in vacuo at room temperature.

### Mass identification of chitobiose by QTOF-MS

Chitobiose obtained from quantitative HPLC was further analyzed by quadrupole-time‐of‐flight‐mass spectrometry (QTOF-MS) (Bruker Biospin AG, Bangkok, Thailand). Chitobiose dissolved in water (2 mg.mL^−1^, 100 μL) was injected into the instrument. A mass range of 50–1000 was selected for data acquisition. Positive ionization mode was chosen using source type Electrospray Ionization (Bruker Apollo II, Thailand). The capillary and charging voltage were set at 4500 V and 2000 V, respectively.

## Results

### Enzyme expression, purification, and chitinase activity assay

**Fig. 1:**
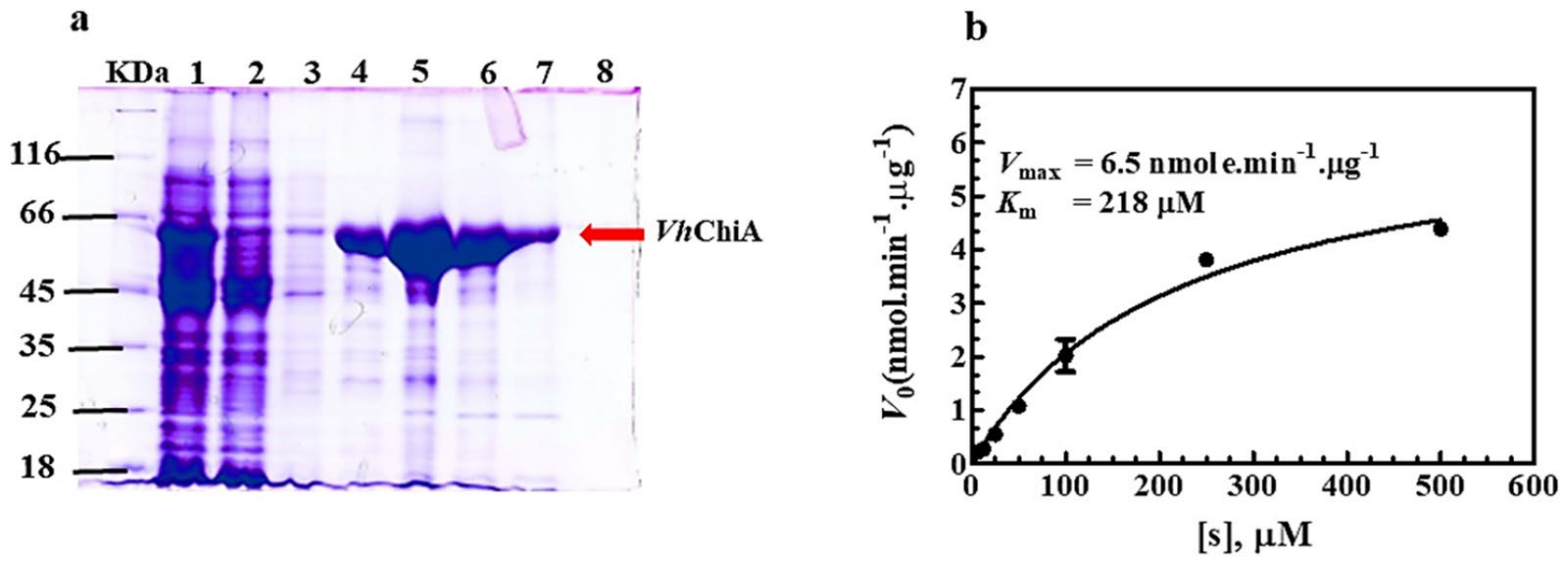
Expression, purification and chitinase activity determination of *Vh*ChiA. **a** SDS-PAGE analysis. *Vh*ChiA was eluted from a Ni–NTA agarose affinity column with 150 mM imidazole in the equilibration buffer and its purity was assessed by 12% SDS-PAGE. kDa, protein markers; Lane 1, crude extract; 2, unbound fraction; 3, fraction washed with the equilibration buffer; 4–7, fractions eluted with 150 mM imidazole; 8, last fraction washed with the equilibration buffer. **b** Michaelis–Menten plot of the initial rate (*v*_0_) *vs. p*NP-(GlcNAc)_2_ concentration [S]

*Vh*ChiA was expressed in *E. coli* M15 (pREP) host cells and purified to > 95% homogeneity using single-step Ni–NTA affinity chromatography. The molecular mass of the purified *Vh*ChiA was estimated to be 63 kDa on SDS-PAGE (Fig. 1a), consistent with that reported previously (Suginta et al. 2004). The enzyme activity in units (U) was calculated from the resultant *V*_max_ of 6.5 nmol min^−1^ 1 μg^−1^ enzyme (Fig. 1b). The kinetic parameters (*V*_max_ = 6.5 nmol.min^−1^.µg^−1^. and *K*_m_ = 216 µM) were obtained from the non-linear regression curve fitted using the Michaelis–Menten equation, available in GraphPad prism *v* 5.01. In a representative enzyme preparation, the total yield of *Vh*ChiA obtained from 8 l of bacterial culture was 380,000 U.

### Enzyme stability

We first examined enzyme stability during the hydrolytic reaction in the absence or presence of BSA, to obtain the greatest yield of sugar product. Enzyme stability was determined at different time intervals of 0, 2.5, 5.0,10.0, 30.0 min and 1.0, 16, and 24 h. Additional file 1: Fig. S1a shows the relative activity of *Vh*ChiA in the absence of BSA. The chitinase activity was found to decrease gradually over the period of incubation time. At 24 h, only 65% the chitinase activity remained. On the other hand, with the addition of BSA (with a 1:2 ratio of *Vh*ChiA:BSA) to the reaction mixture full chitinase activity was retained throughout 24 h of incubation (Additional file 1: Fig. S1b).

### Substrate specificity of *Vh*ChiA

Avicel® microcrystalline cellulose, mannan, partially de-acetylated chitin (chitosan) and colloidal chitin were used to examine the specificity of *Vh*ChiA. As shown in Additional file 1: Fig. S2. *Vh*ChiA hydrolyzed chitin and partially de-acetylated chitin but did not hydrolyze non-chitin polysaccharides (cellulose and mannan). There was no detectable product from reaction mixes containing microcrystalline cellulose (Additional file 1: Fig. S2a) or mannan (Additional file 1: Fig. S2b) at different incubation times up to 24 h. For mannan, the pale spot observed at the position close to DP5 was likely to be an artifact as it was also observed in the control reaction. When partially de-acetylated chitin was used as substrate (Additional file 1: Fig. S2c), no product was observed within 1 h, but three spots, corresponding to (GlcNAc)_2_, (GlcNAc)_3_ and (GlcNAc)_5_, appeared after 16 h and 24 h of reaction. With chitin as substrate (Additional file 1: Fig. S2d), no reaction product was seen within 1 h, but there were strong spots, corresponding to (GlcNAc)_2_ and (GlcNAc)_3_, after 16 h and 24 h of reaction. Intensities of the sugar spots on the TLC plates were further analyzed and peak areas obtained from ImageJ analysis showed that colloidal chitin was the preferred substrate, followed by partially deacetylated chitin. Table 1 summarizes the quantification of the reaction products from enzymic cleavage of different polysaccharides using *Vh*ChiA. Analysis of peak intensities yielded a peak area for (GlcNAc)_2_ of 3480 a.u. and for (GlcNAc)_3_ of 2200 a.u., corresponding to overall yields of 61.0% and 39.0%, respectively, from chitin (Additional file 1: Fig. S2d). At the same time point, partially deacetylated chitin or simple chitosan (Additional file 1: Fig. S2c) was cleaved to (GlcNAc)_2_ (peak area = 2,800 a.u.), (GlcNAc)_3_ (peak area = 2,600 a.u.) and (GlcNAc)_5_ (peak area = 2,600 a.u.), with overall yields of 35%, 33% and 33%, respectively.

**Table 1:** Quantitation of chitooligosaccharide products obtained from the hydrolysis of different polysaccharides by *Vh*ChiA

Substrate	Hydrolytic product					
	(GlcNAc)_2_		(GlcNAc)_3_		(GlcNAc)_5_	
	Peak area (a.u.)^a^	% yield	Peak area (a.u.)^a^	% yield	Peak area (a.u.)^a^	% yield
Microcrystalline cellulose	0	0	0	0	0	0
Mannan	0	0	0	0	0	0
Partially deacetylated chtin	2800	35	2600	33	2600	33
Colloidal chitin	3480	61	2200	39	0	0

The intensities of migrating sugar spots were measured after 24 h of reaction for each polysaccharide, using ImageJ software (https://imagej.nih.gov/ij/)

^a^a.u. represents arbitrary unit

### Production of chitobiose from different forms of chitin

**Fig. 2:**
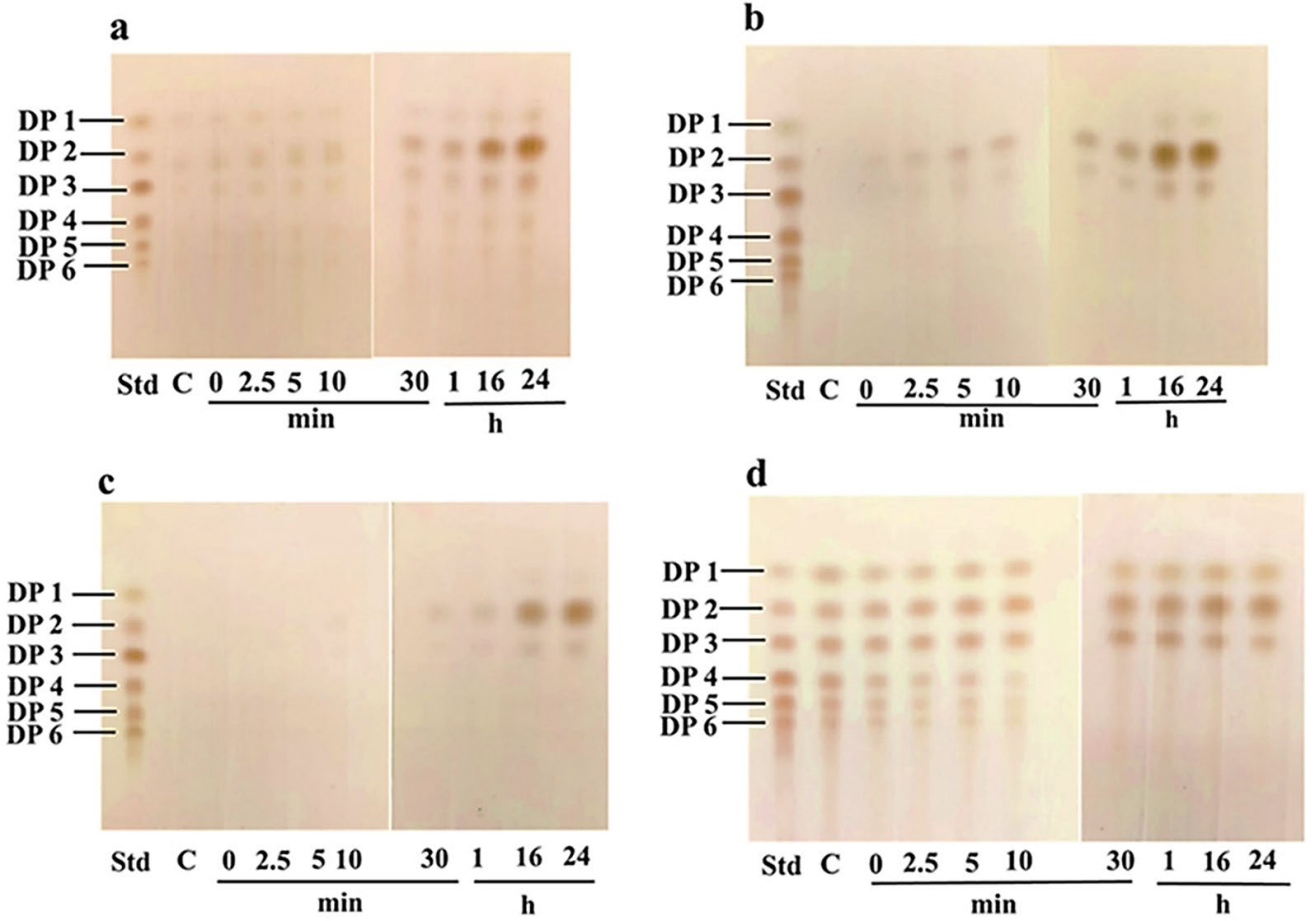
Time-course of hydrolysis of various types of chitins by *Vh*ChiA. **a** β-chitin (squid pen), **b** α-chitin (shrimp shell), **c** α-chitin (crab shell) and **d** crude chitooligosaccharide mixture. Small aliquots of the reaction sample from time points 0, 2.5, 5, 10, 30 min and 1,16, 24 h were analyzed by TLC. Each reaction mix contained 5 mg (dry weight) of different colloidal chitins in 0.1 M sodium acetate buffer pH 5.5 and 100 U of *Vh*ChiA. The control was substrate alone. Std represents a mixture of (GlcNAc)_1–6_ (labeled DP1–DP6), migrating alongside the reaction samples. Control lanes (C) contained the substrate but no enzyme

The time-course study showed that *Vh*ChiA hydrolyzed chitin, yielding different sizes of chitooligosaccharides as reaction intermediates, and at the end of the reaction (GlcNAc)_2_ was the main product. Figure 2 shows the TLC analysis of the reaction products obtained after different times of hydrolysis of squid pen chitin (Fig. 2a), shrimp chitin (Fig. 2b) and crab chitin (Fig. 2c), respectively. The standard CHO mixture, containing (GlcNAc) to (GlcNAc)_6_ (labeled DP1 − DP6), was applied alongside the reaction products from 0 min to 24 h. Figure 2a shows that *Vh*ChiA could hydrolyze squid pen chitin, with faint spots corresponding to (GlcNAc)_2_ and (GlcNAc)_3_ in the early stages of the reaction (0–30 min), The spot of the main product (GlcNAc)_2_ became greater with progress of the reaction. Hydrolysis of shrimp chitin (Fig. 2b) and crab chitin (Fig. 2c) yielded similar results: no reaction product was seen at 0–10 min, while faint bands of (GlcNAc)_2_ and (GlcNAc)_3_ appeared at 30 min to 1 h and the intensity of both sugars increased with progress of the reaction, and as observed before (GlcNAc)_2_ was the exclusive product after 24 h of reaction. Figure 2d shows the hydrolysis of a chitooligosaccharide mixture by *Vh*ChiA. The hydrolysis products detected within 10 min were GlcNAc–(GlcNAc)_6_, but when the reaction was continued to 24 h, GlcNAc, (GlcNAc)_2_ and (GlcNAc)_3_ were the main products.

### Analysis of hydrolytic products by quantitative HPLC

**Fig. 3:**
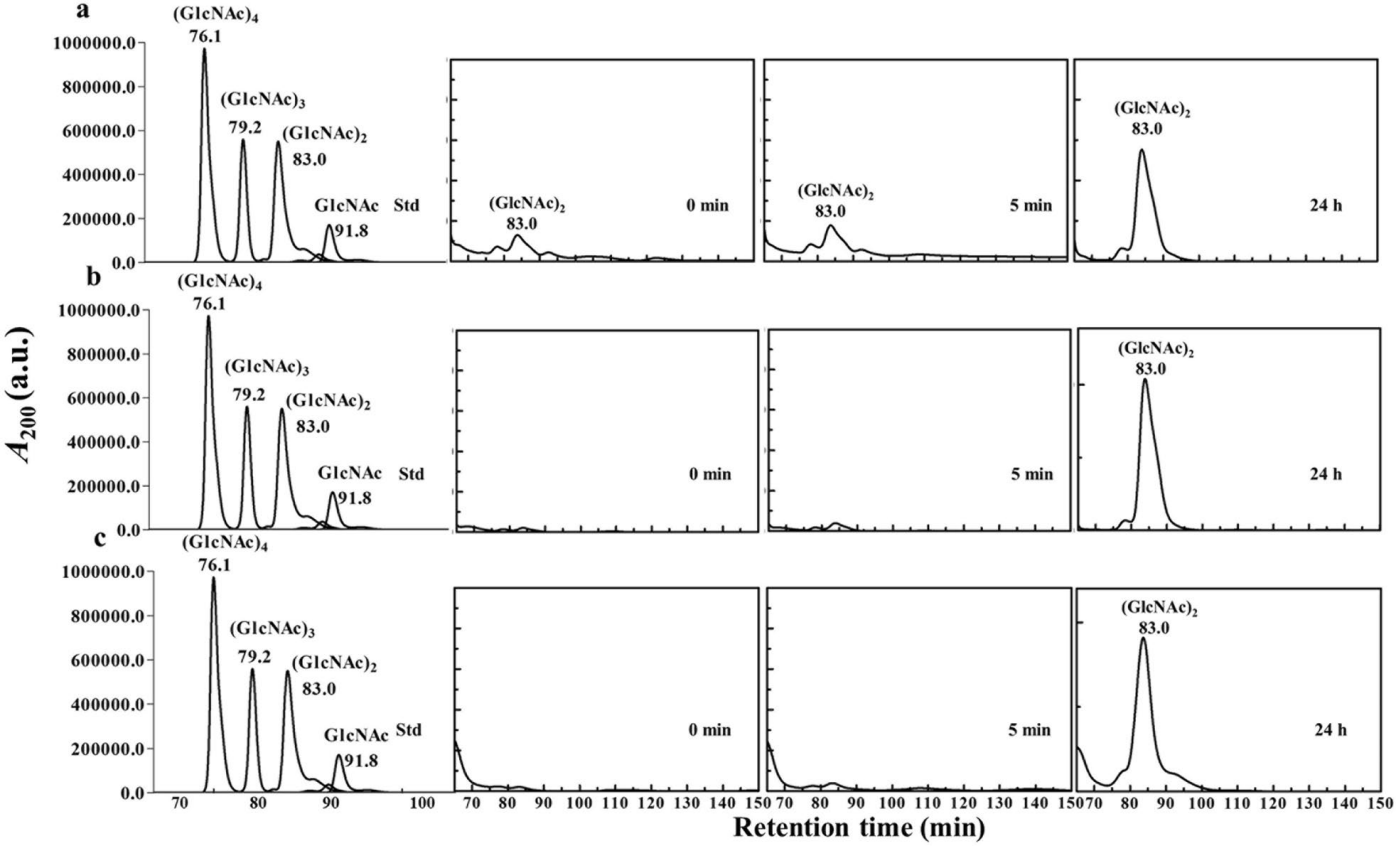
HPLC analysis of chitin hydrolysis by *Vh*ChiA. **a** Squid pen chitin, **b** shrimp chitin, **c** crab chitin. Each reaction sample of 20 μL (carried out as described in text) was applied to a TSK Gel G2000 PW column connected with a Shimadzu HPLC system. Left panels are standard chitosugars (GlcNAc) – (GlcNAc)_4_ run under the same conditions as the reaction samples

For quantitative analysis of chitin degradation by *Vh*ChiA, each reaction sample obtained after different time intervals was analyzed by size exclusion chromatography using a TSK Gel G2000 PW column (7.5 mm × 30 cm) connected to an HPLC system (Shimadzu, Thailand). Figure 3 shows the size exclusion profiles of the reaction products obtained at 0 min, 5 min and 24 h. In general, similar profiles were observed for squid pen chitin (Fig. 3a), shrimp chitin (Fig. 3b) and crab chitin (Fig. 3c), from which small amounts of GlcNAc, (GlcNAc)_2_, and (GlcNAc)_3_ were formed as the primary products at 5 min of reaction, with (GlcNAc)_2_ being the major product. The formation of (GlcNAc)_2_ increased with all substrates and reached its highest level at 24 h.

**Fig. 4:**
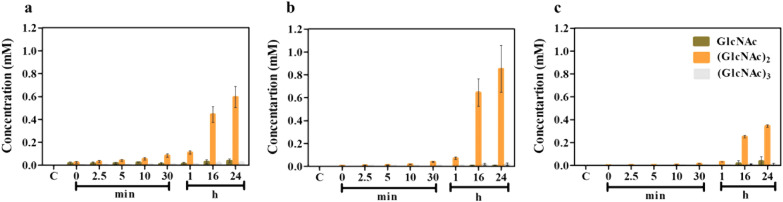
Quantitative analysis of hydrolysis products by HPLC. Substrates were **a** squid pen, **b** shrimp shell and **c** crab shell chitin, shown as bar graphs of concentration against the reaction time. Concentrations were estimated from the corresponding standard calibration curve, for the three main hydrolytic products GlcNAc, (GlcNAc)_2_ and (GlcNAc)_3_). The control (C) contained the substrate but no enzyme. Values are means ± SD

Figure 4 shows bar graphs of the changes in the concentrations of the reaction products (GlcNAc), (GlcNAc)_2_ and (GlcNAc)_3_ after different times of incubation. After 16 and 24 h of incubation, (GlcNAc)_2_ was the exclusive product from squid chitin (Fig. 4a), shrimp chitin (Fig. 4b) and crab chitin (Fig. 4c). The highest yield of (GlcNAc)_2_ was obtained from shrimp chitin (0.85 ± 0.20 mM), followed by squid pen chitin (0.60 ± 0.10 mM) and crab chitin (0.34 ± 0.01 mM).

The overall yields of chitooligosaccharide products obtained during a small-scale production from three different chitins are summarized in Table 2. The purity of (GlcNAc)_2_ obtained from shrimp-shell chitin was 96% and 91% from squid pen chitin and crab-shell chitin, while GlcNAc and (GlcNAc)_3_ were minor products and the purity of both products was < 10%. The results obtained from the small-scale trials suggested that shrimp chitin is the best substrate for chitobiose production by *Vh*ChiA.

**Table 2:** A summary of product yield (mg) of colloidal chitin degradation by *Vh*ChiA

Substrate	GlcNAc		(GlcNAc)_2_		(GlcNAc)_3_	
	% purity	mg	% purity	mg	% purity	mg
Shrimp chitin	0.2	0.01 ± 0.01	96	1.8 ± 0.4	4.2	0.10 ± 0.04
Squid chitin	2.4	0.05 ± 0.02	91	1.3 ± 0.2	6.8	0.10 ± 0.02
Crab chitin	3.7	0.06 ± 0.07	91	0.8 ± 0.1	5.7	0.04 ± 0.02

The % purity for each hydrolytic product was calculated from the individual peak area divided by the total peak area obtained from all the products in the HPLC chromatogram. Values are means ± SD from two independent reactions

**Fig. 5:**
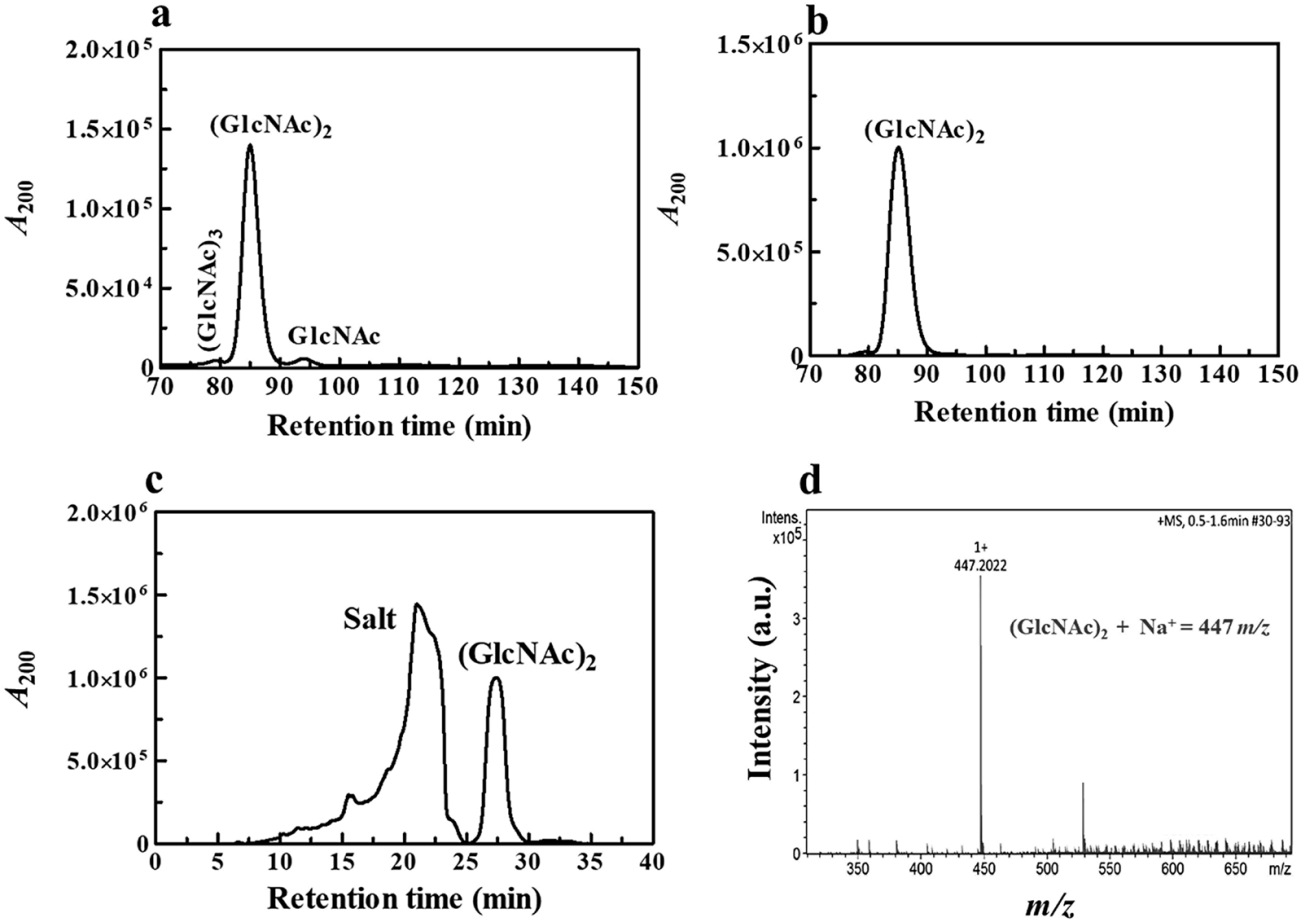
Purification of (GlcNAc)_2_ product. **a** Reaction sample before passage through a gel filtration column packed with cellulose beads (Cellufine, lot No. R2407, JNC Corporation, Japan) column. **b** Reaction sample after gel filtration. Sugar products were detected at 200 nm. DP1, DP2, and DP3 represent GlcNAc, (GlcNAc)_2_ and (GlcNAc)_3_, respectively. **c** (GlcNAc)_2_ was eluted from a preparative NH_2_P-50 10E column, by acetonitrile water mixture (70:30 *v/v*) and detected in a PDA mode 200 nm. The retention time of the product was compared with those of standard sugars (data not shown). **d** Product characterization of chitobiose by QTOF-MS

We further attempted larger scale production of chitobiose. The hydrolysis of 1 g of shrimp colloidal chitin yielded (GlcNAc)_2_ as the major product, with small amounts of GlcNAc and (GlcNAc)_3_ (Fig. 5a), shown before the gel filtration step. When the reaction products were analyzed by TLC, only (GlcNAc)_2_ was observed, while the other two products were not detected due to their low concentration in the samples. The reaction products were further analyzed by HPLC (TSK Gel G2000 PW column, 7.5 mm × 30 cm), and from their corresponding peak areas, the purities of GlcNAc, (GlcNAc)_2_ and (GlcNAc)_3_ were calculated to be 96.2% for (GlcNAc)_2_, 2.3% for GlcNAc and 1.5% for (GlcNAc)_3_. After further purification on a gel filtration column (2.1 cm × 160 cm) packed with cellulose beads (Cellufine, Lot No. R2407, JNC Corporation, Japan), the final purity of (GlcNAc)_2_ was increased to 99% with an apparent yield of 3.6 g from 1 g of the starting material (Fig. 5b). Since the apparent yield of the final product exceeded the initial substrate quantity after the gel filtration step, we assumed the presence of salt contamination in the chitobiose product, and the sugar was therefore further purified using an Asahipak NH2P-50 10E preparative column (10.0 mm × 250 mm, Shodex China Co., Ltd), connected to HPLC. The HPLC separation indicated two resolved peaks, the earlier peak being salt, while the latter was eluted in the position of chitobiose ((labeled DP2) (Fig. 5c). The final yield obtained after elimination of salt was 200 mg of purified chitobiose, with purity > 99%. The summary of the chitobiose preparation is presented in Table 3. In order to characterize the composition of the final product, the purified chitobiose was injected into QTOF-MS. The analyzed sample yielded the major mass with m/z of 447.2, which corresponded to the ionized mass of (GlcNAc)_2_ + Na^+^ (Fig. 5d).

**Table 3:** A summary of production of (GlcNAc)_2_ from 1 g of shrimp chitin

Substrate	(GlcNAc)_2_ product		
	Before gel filtration	After gel filtration	Preparative HPLC
Shrimp chitin	4.3 g	3.6 g	0.2 g
(α-form)	salt + GlcNAc + (GlcNAc)_2_ + (GlcNAc)_3_	salt + (GlcNAc)_2_	(GlcNAc)_2_

(a) Before gel filtration, (b) after gel filtration. The reaction sample (20 μL) was injected into the TSK Gel G2000 PW column and resolved as described in text. (c) Removal of salt from the (GlcNAc)_2_. The chitobiose salt mix obtained after gel filtration step was further injected into the NH_2_P-50 10E preparative column. Chitobiose fractions were eluted with an acetonitrile:water mixture (70:30 *v/v*), with a flow rate 1 mL.min^−1^ and detected by PDA detector at 200 nm

## Discussion

Chitin is the most abundant polysaccharide in oceans and recycling of crustacean chitins is naturally achieved by marine bacteria, especially those of the *Vibrio* class (Keyhani and Roseman 1999). Such marine bacteria usually secrete several chitinolytic enzymes, for instance lytic polysaccharide monooxygenase **(**Vaaje-Kolstad et al. 2010), chitinases (Bhattacharya et al. 2007; Rathore and Gupta 2015; Suginta et al. 2005, 2000) and *N*-acetylglucosaminidases (Sahai and Manocha 1993; Suginta et al. 2000; Sirimontree et al. 2014), and then utilize the degradation products (CHOs) as their energy source. Breakdown by chitinases is considered to be the key extracellular step in chitin degradation. Numerous chitinases have been cloned and characterized intensively, but the exploitation of chitinases in the production of CHOs from chitin wastes is still limited. Most studies have reported proof-of-concept data in small-scale reactions with milligrams of chitin substrate, and the sugar products were mostly not further purified or characterized. We previously reported that the chitinase isoforms Chi-90 and Chi-65 from *V. alginolyticus* 283 could digest chitin, generating chitobiose as the major product (approximately 95% yield) (Suginta 2007). More extensive studies were performed with chitinase A from *Vibrio campbellii* (formerly *V. harveyi*) type strain ATCC BBA 1116 **(**Suginta et al. 2000, 2004; Suginta 2007; Songsiriritthigul et al. 2008). *Vh*ChiA was well expressed, easily purified, relatively stable, had a high turnover rate (*k*_cat_ = 7.4 ± 0.5 s^−1^) with *p*NP-(GlcNAc)_2_ as substrate and proved to be a potent biocatalyst for chitobiose production. Our laboratory-scale trials showed that we could conveniently produce some several hundred milligrams of highly purified chitobiose in a single-step reaction. Comparing chitin from shrimp, crab and squid pen as starting materials, the highest yield was from shrimp chitin. The greatest advantage of our method is that the purity of the product, chitobiose, is > 99%, which is greater than that currently available commercially. Table 4 summarizes CHO production using enzymic hydrolysis of chitin from different microbial organisms.

**Table 4:** A summary of chitobiose production by different microbial enzymes

Substrate	Scale	Enzyme Source	Product	Yield (GlcNAc)_2_	Purity	Analytical method	References
Swollen chitin (Crab shell)	3 mg^a^	*Aeromonas* sp GJ-18	(GlcNAc)_1–3_	1.1 mg^a^	n.d	HPLC	(Kuk et al. 2005)
Swollen chitin	5 mg^a^	*Enterobacter sp. NRG4*	(GlcNAc)_1–2_	3.6 mg^a^	n.d	TLC and HPLC	(Kumar et al. 2011)
Colloidal chitin (Crab shell)	1.5 g	*Paenicibacillus barengoltzii*	(GlcNAc)_2_	21.6 mg	99%	TLC and HPLC	(Yang et al. 2016)
Colloidal chitin (Shrimp shell)	2 mg^a^	*Salinivibrio* BAO-1801	(GlcNAc)_1–2_	1.4 mg	n.d	HPLC	(Le and Yang 2018)
Colloidal chitin (Shrimp powder)	2 g^a^	*Streptomyces sampsonii* *XY* 2–7	(GlcNAc)_1–2_	2.2 mg	n.d	TLC and HPLC	(Zhang et al. 2020)
Colloidal chitin (Shrimp/ squid pen)	15 mg	*Chitiniphilus shinanonensis*	(GlcNAc)_1–2_	4.8 mg^a^	n.d	HPLC	(Rani et al. 2020)
Colloidal chitin (α, β)	5 mg	*Paenibacillus sp* LS 1	(GlcNAc)_1–2_	0.2 mg, α-chitin 0.9 mg, β-chitin	n.d	HPLC	(Mukherjee et al. 2020)
Colloidal chitin (Shrimp shell)	1 g	*Thermomyces lanuginosus*	(GlcNAc)_2–3_	100 mg	Partially purified	TLC and HPLC	(Kumar et al. 2021)
Colloidal chitin (Shrimp flakes)	1 g	*Vibrio campbellii*	(GlcNAc)_1–3_	200 mg	> 99%	TLC, HPLC and Q-TOF–MS	This study

^a^Weight is converted from the concentration (% or mM) and reaction volume given in each report

When compared with similar or higher levels of chitin starting materials (1 to 2 g), our chitinase generated a higher yield of chitobiose, being twofold greater than the yield produced by chitinase from *Thermomyces lanuginosus* (Kumar et al. 2021), tenfold greater than with chitinase from *Paenicibacillus barengoltzii* **(**Yang et al. 2016), and 100-fold greater than with chitinase from *Streptomyces sampsonii* XY 2–7 (Zhang et al. 2020). The chitobiose produced from *Vh*ChiA obtained after preparative HPLC was of analytical grade, with a purity of > 99%, which was greater than the purity of chitobiose obtained from all previous studies.

As mentioned earlier, bioconversion of the abundantly available chitin food wastes into CHOs with enriched biological activities has received much attention recently. This study offers an enzyme technology using in-house produced chitinase for the rapid production, on a commercial scale, of high-quality chitobiose from chitin food wastes. The method developed can be applied in industrial processes to obtain the large-scale production of chitobiose with higher purity than the product currently available commercially and is a convenient technique that simplifies the difficult process of CHO production. Despite the versatile properties of chitin, studies related to the synthesis of various derivatives are limited due to the insufficient availability of the starting material, in particular chitobiose. Therefore, this study offers the possibility of producing a sufficient quantity of chitobiose, which can be used to synthesize other chitin derivatives.

## Future perspectives

CHOs have broad range of applications due to their water solubility, and also possess useful biological properties, including anti-microbial, anti-tumor and anti-oxidative activities. Since pure standard sugars are quite expensive and difficult to obtain in large-scale at present, it is essential to develop a novel method of producing CHOs with specific degree of polymerization. Chitobiose obtained from this study can be used as a starting material for chemo-enzymic synthesis of various CHO derivatives, for instance GlcNAc-GlcN, *p*NP-(GlcNAc)_2_, 4MU-(GlcNAc)_2_ and other functionalized CHO-based nanomaterials. It can also be used to produce high-molecular weight CHOs through transglycosylation reactions. It is stated that for the efficient production of sugar, pretreatment of chitin requires chemical degradation, followed by the enzymic hydrolysis. Pretreatment of chitin using ionic liquids is an alternative to chemical treatment, but the cost of ionic liquids is high, which is an obstacle to their use. Chitobiose can also be used as a starting material for the synthesis of rare sugars, for example heterogenous sugars such as (GlcNAc-GlcN) or (GlcN-GlcNAc) using deacetylase enzymes, or for the synthesis of different types of chitooligosaccharide derivatives.

### Supplementary Information

**Additional file 1: Fig. S1.** The retained activity of VhChiA against pNP-(GlcNAc)2 at 30 °C without added BSA (a) and with BSA (b). The release of pNP was monitored by light absorption at 405 nm and this value was converted into relative activity (set as 100% for full activity). Values shown are means ± SD. **Fig. S2.** Time-course of hydrolysis of different types of polysaccharides by VhChiA. A standard mixture of CHOs (lane: std) was applied together with the reaction sample. (a) Avicel®crystalline cellulose, (b) mannan, (c) partially deacetylated chitin (chitosan), (d) squid pen chitin. Control (C) contained the substrate with no enzyme. The reaction was carried out for different time intervals: 0, 2.5, 5, 10, 30 min, and 1, 16, 24 h and the reaction was stopped by boiling at 98 °C for 5 min.

## Data Availability

All data can be supplied upon request.

## Abbreviations

CHOs: Chitooligosaccharides
TLC: Thin layer chromatography
(GlcNAc)_n_: N = 2, 3, 4…, chitobiose, chitotriose, chitotetraose, …
*p*NP-(GlcNAc)_2_: 4-Nitrophenyl *N,N*-diacetyl-β-D-chitobioside
