# Supplementary material for: Bioeconomic production of high-quality chitobiose from chitin food wastes using an in-house chitinase from Vibrio campbellii
Source: Bioresour Bioprocess. 2022 Aug 20;9(1):86. doi: 10.1186/s40643-022-00574-8 (PMC10991452; doi:10.1186/s40643-022-00574-8)
Supplement: Supplementary file 1 — Additional file 1: Fig. S1. The retained activity of VhChiA against pNP-(GlcNAc)2 at 30 °C without added BSA (a) and with BSA (b). The release of pNP was monitored by light absorption at 405 nm and this value was converted into relative activity (set as 100% for full activity). Values shown are means ± SD. Fig. S2. Time-course of hydrolysis of different types of polysaccharides by VhChiA. A standard mixture of CHOs (lane: std) was applied together with the reaction sample. (a) Avicel®crystalline cellulose, (b) mannan, (c) partially deacetylated chitin (chitosan), (d) squid pen chitin. Control (C) contained the substrate with no enzyme. The reaction was carried out for different time intervals: 0, 2.5, 5, 10, 30 min, and 1, 16, 24 h and the reaction was stopped by boiling at 98 °C for 5 min. [file 40643_2022_574_MOESM1_ESM.docx]

**Bioeconomic production of highly quality chitobiose from chitin food wastes using a chitinase from *Vibrio campbellii***

**Reeba Thomas^a^, Tamo Fukamizo,^a^ Wipa Suginta^a*^**

^a^ School of Biomolecular science and Engineering (BSE), Vidyasirimedhi Institute of Science and Technology (VISTEC), Payupnai, Wangchan District, Rayong, Thailand 21210

**Corresponding author:** Prof. Wipa Suginta, Vidyasirimedhi Institute of Science and Technology, Rayong, 20210, Thailand

**Running title**

Enzymatic production of chitobiose from chitin biomass

**Corresponding author:** Wipa Suginta, Vidyasirimedhi Institute of Science and Technology (VISTEC), Rayong, 20210, Thailand, Email: [wipa.s@vistec.ac.th](mailto:wipa.s@vistec.ac.th)

**Supplemental materials**

**a**

**b**


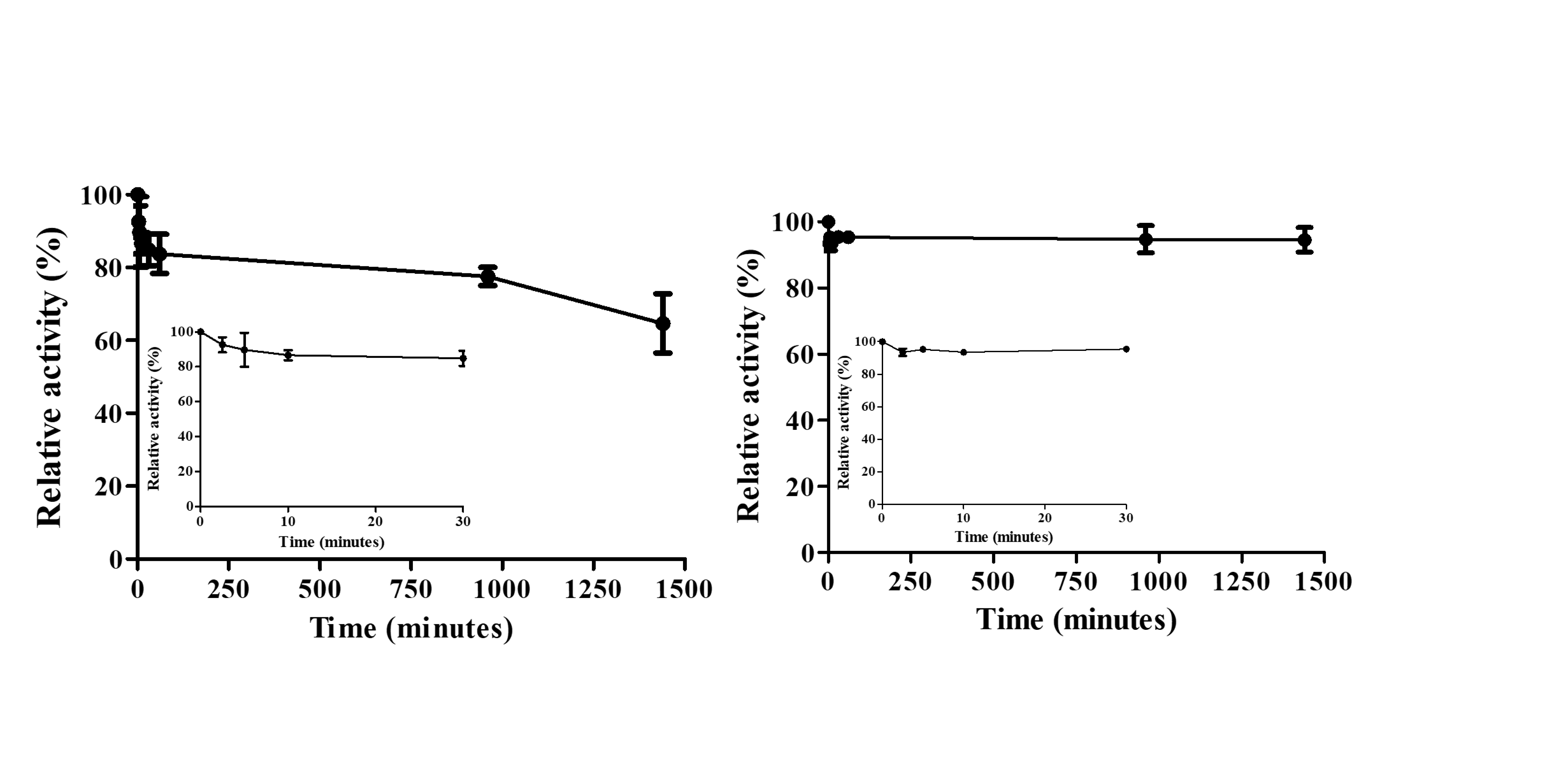


**Fig. S1:** The retained activity of *Vh*ChiA against *p*NP-(GlcNAc)_2_ at 30°C without added BSA (a) and with BSA (b). The release of *p*NP was monitored by light absorption at 405 nm and this value was converted into relative activity (set as 100% for full activity). Values shown are means ± SD.


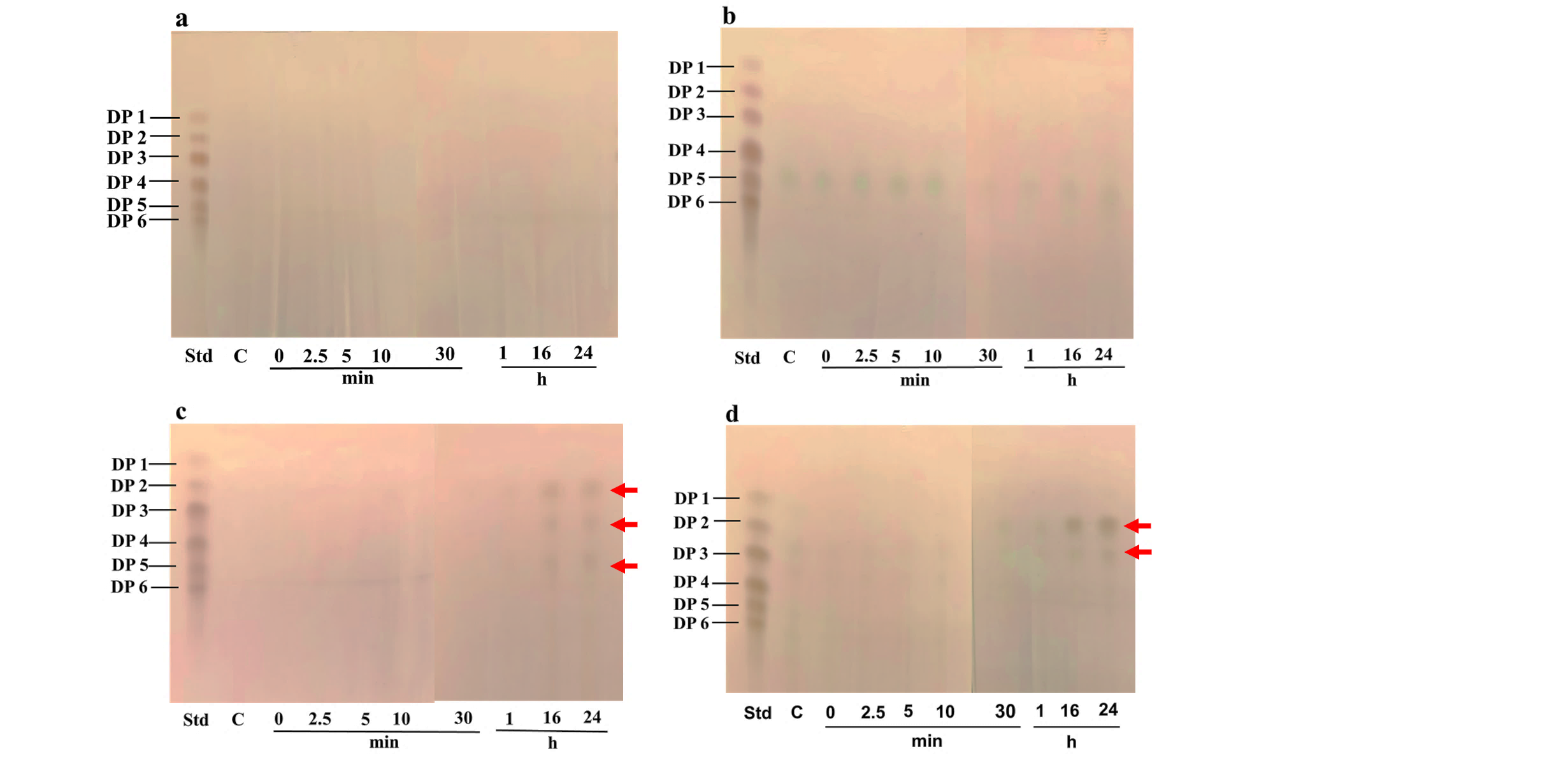


**Fig. S2:** Time-course of hydrolysis of different types of polysaccharides by *Vh*ChiA. A standard mixture of CHOs (lane: std) was applied together with the reaction sample. (a) Avicel®crystalline cellulose, (b) mannan, (c) partially-deacetylated chitin (chitosan), (d) squid pen chitin. Control (C) contained the substrate with no enzyme. The reaction was carried out for different time intervals: 0, 2.5, 5, 10, 30 min, and 1, 16, 24 h and the reaction was stopped by boiling at 98 °C for 5 min.
